# Developing the Readout Electronics for a Custom 64 × 64 SPAD Array: From Single-Board Prototyping to FPGA Implementation Toward Stellar Intensity Interferometry

**DOI:** 10.3390/s26185757

**Published:** 2026-09-10

**Authors:** Álvaro Quintana, Guillermo González-de-Rivera, Sergio López-Buedo, Francisco Prada

**Affiliations:** 1 Hardware and Control Technology Laboratory (HCTLab), Universidad Autónoma de Madrid, 28049 Madrid, Spain; guillermo.gdrivera@uam.es; 2 High Performance Computing and Networking (HPCN), Universidad Autónoma de Madrid, 28049 Madrid, Spain; sergio.lopez-buedo@uam.es; 3 Instituto de Astrofísica de Andalucía (IAA), Consejo Superior de Investigaciones Científicas (CSIC), 18008 Granada, Spain; f.prada@csic.es

**Keywords:** SPAD array, single-photon detection, intensity interferometry, astrophysics, FPGA, Zynq UltraScale+ MPSoC, Raspberry Pi, address-event representation (AER), White Rabbit, dark-count rate (DCR)

## Abstract

Single-photon avalanche diode (SPAD) arrays enable photon-starved applications, including time-of-flight imaging and stellar intensity interferometry. Their astronomical use remains scarcely explored, as the bottleneck is usually not detection but rather acquisition electronics for high-rate event streams. This work presents a modular, event-driven acquisition system for a 64 × 64 SPAD array within the La Palma Quantum Interferometer (LPQI) project, repurposing a LiDAR detector for multi-telescope interferometry. Two stages are used: a Raspberry Pi 5 with a custom board for validation, and an AMD Kria KR260 (Zynq UltraScale+ MPSoC) implementing the Address-Event Representation (AER) handshake in hardware at 100 MHz. The system streams AER events without per-event timestamping; sub-nanosecond time-tagging is left for a future stage based on the White Rabbit protocol. Optical bench tests confirmed spatial detection and localization of photons at a measured throughput of up to ≈124 keps under the highest illumination condition tested, and dark-count-rate characterization showed a rate below 10 Hz for most pixels (median: 1.68 Hz at 27.8 °C); raw per-pixel event-count maps further confirmed, for the first time on this array, the expected 2 × 2 spatial pattern of inter-pixel crosstalk from its shared-cathode pixel groups. The results demonstrate the feasibility of repurposing a LiDAR SPAD sensor and establish an acquisition-electronics baseline to aid the development of a timestamped, multi-telescope system for deployment on five telescopes of the Roque de los Muchachos Observatory.

## 1. Introduction

Single-photon avalanche diode (SPAD) arrays are a state-of-the-art photodetection technology capable of registering single-photon events with a temporal resolution on the order of picoseconds [1]. Their adoption in applications such as time-of-flight (ToF) LiDAR, 3D imaging, quantum communications, and biomedical imaging is well established [2,3,4,5,6,7,8]. However, their use in astronomical instrumentation, particularly for intensity interferometry in the optical domain, remains very limited and is barely explored in the recent literature [9,10].

Intensity interferometry achieves levels of angular resolution much higher than those of current telescopes by correlating the intensity fluctuations detected simultaneously by spatially separated telescopes [11,12]. Unlike amplitude interferometry, it does not require preserving the phase coherence of the wavefront, making it robust against atmospheric turbulence. However, intensity interferometry in the optical domain poses many technological challenges: both the timestamping of photon arrivals and the temporal synchronization between telescopes need to be extremely precise (up to the picosecond level), and the readout electronics should be able to manage data flows of several million events per second. As a result, although intensity interferometry in the optical domain is a very promising technique for astronomy, it remains at a very early stage of development.

In principle, SPAD arrays are well suited to intensity interferometry because of their high sensitivity, ultrafast temporal resolution, and advanced fabrication processes for both commercial and industrial applications. However, most SPAD arrays developed for LiDAR and 3D imaging integrate a time-to-digital converter (TDC) at the pixel or column level and expose the resulting timestamps or histograms through a synchronous digital interface [4,6,8]. Although this approach matches the requirements of range applications well, it is not directly compatible with the low-latency, node-to-node event correlation that intensity interferometry requires.

Recently, there has been interest in image sensors using the Address-Event Representation (AER) protocol [13]. The main benefit of the AER protocol is that it allows direct connection of the sensor to neuromorphic image-processing systems based on spiking neural networks [14,15]. Additionally, since the AER protocol is fully asynchronous and events are only transmitted when they occur, it also allows for power-efficient interfaces. In the context of image sensors, AER events correspond to a pixel reaching a certain threshold. In the case of SPAD arrays, AER events correspond to a photon triggering an avalanche at a pixel photodetector. This asynchronous, event-driven behavior is precisely what makes the AER protocol attractive for interferometry since photon arrivals could be forwarded with minimal latency to the readout electronics.

**Figure 1:**
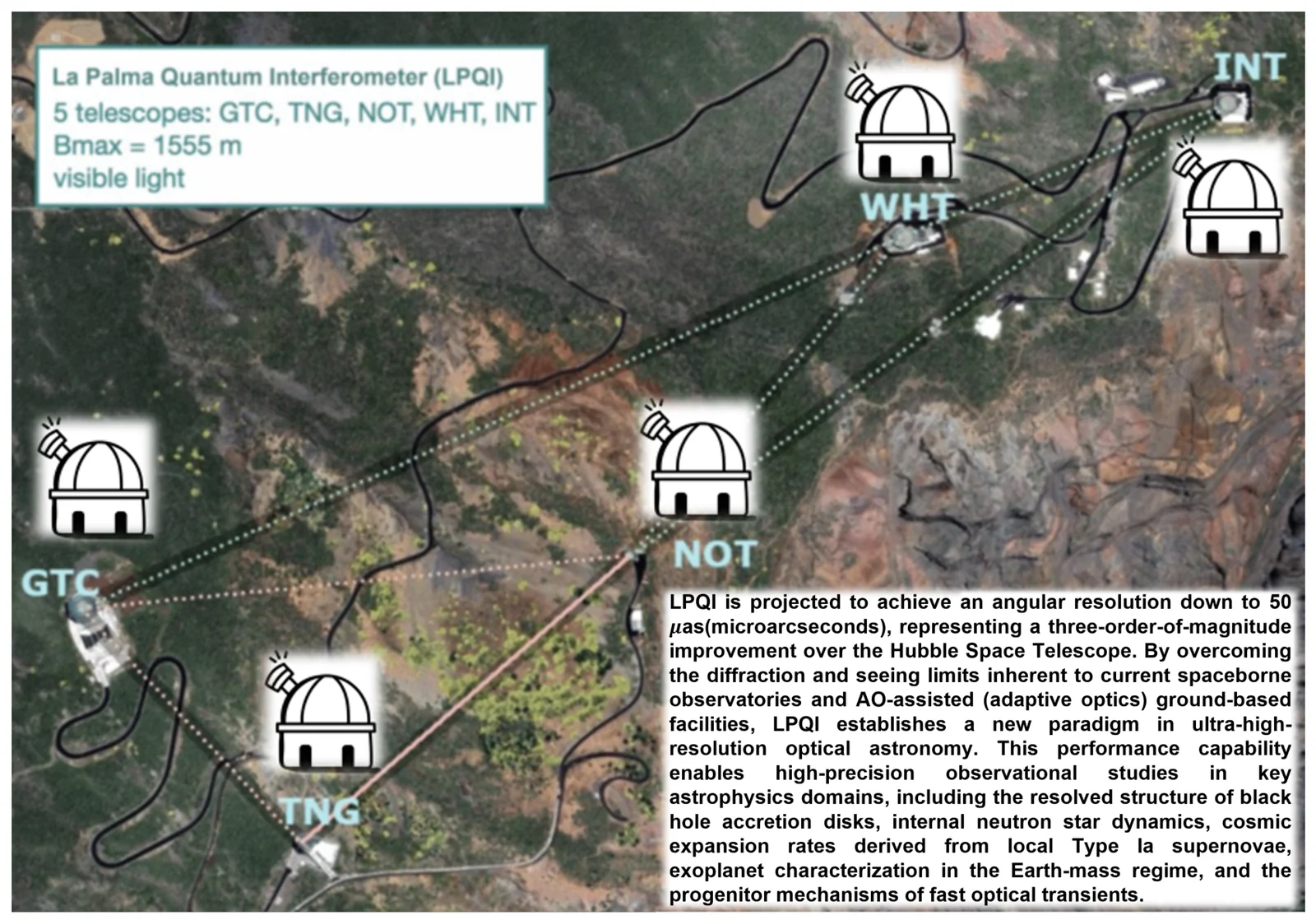
La Palma Quantum Interferometer (LPQI) telescope array at Roque de los Muchachos Observatory, Canary Islands (Spain).

In this paper, we investigate the use of an SPAD array with an AER interface developed for research purposes for intensity interferometry [2,16]. Although the sensor was originally developed for LiDAR applications, the combination of SPAD technology with the AER protocol makes it a strong candidate for intensity interferometry. However, since conventional acquisition electronics are designed around synchronous sensor interfaces, it is first necessary to develop custom readout electronics able to handle an asynchronous event stream at multi-million-events-per-second rates. Here, we present a proof of concept toward such readout electronics, developed in the context of the La Palma Quantum Interferometer (LPQI) project [17,18] (Figure 1), with the aim of temporally correlating stellar photons detected across the Roque de los Muchachos Observatory’s telescopes. In its first phase, the LPQI-Pathfinder will correlate photon detections between the Nordic Optical Telescope (NOT) and the Telescopio Nazionale Galileo (TNG), separated by 550 m; a planned extension incorporating the Gran Telescopio Canarias (GTC), the William Herschel Telescope (WHT), and the Isaac Newton Telescope (INT) will increase the interferometric baselines to 1.5 km.

The proposed methodology for developing the readout electronics combines an initial, software-based prototyping stage on a Raspberry Pi 5 with a secondary, hardware-based stage on a COTS (commercial, off-the-shelf) FPGA platform: the AMD Kria KR260. This methodology allows quick evaluation of the SPAD array and rapid development of the configuration software, paving the way for the final FPGA implementation, which handles the AER interface at maximum speed. In this way, we demonstrate that this asynchronous, research-grade SPAD array can be adapted to the astronomical domain through modular, scalable acquisition electronics, without redesigning the sensor itself. This study’s contributions are as follows:

– We designed a modular, two-stage readout electronics architecture, comprising a Raspberry Pi 5 prototyping stage and an AMD Kria KR260 (based on Zynq UltraScale+ MPSoC) final stage, which share the same conditioning PCB thanks to an industry-standard 40-pin interface, which enables an incremental transition from a software-driven to a fully hardware-driven AER handshake. To the best of our knowledge, this is the first readout electronics system of this kind developed specifically to adapt an event-driven, AER-based SPAD array to the throughput and modularity requirements of astronomical intensity interferometry.
– We present a complete hardware implementation of the AER-to-AXI4-Stream/DMA acquisition pipeline, verified at clock-cycle resolution with an Integrated Logic Analyzer and characterized in terms of timing margin, logic and memory utilization, and on-chip power consumption.
– We provide experimental validation on an optical bench, including spatial localization of photon events under variable illumination levels and a thermally resolved characterization of the dark-count rate, providing baseline noise figures relevant to future photon-correlation measurements.
– We identify the current system bottlenecks, most notably the absence of per-event timestamping in the present implementation, and formulate a concrete technical roadmap toward multi-telescope deployment, offering guidance that is transferable to other groups seeking to adapt commercially or industrially developed SPAD sensors to scientific instrumentation.

The remainder of this paper is organized as follows. Section 2 reviews SPAD technology and intensity interferometry. Section 3 describes the two-stage system architecture and its implementation, including the FPGA acquisition pipeline, the conditioning board, and its resource usage. Section 4 demonstrates experimental validation, and Section 5 provides our conclusions and outlines future work.

## 2. Background

### 2.1. SPAD Technology Overview

SPAD arrays consist of a grid of photodetectors in which each pixel operates in Geiger mode, reverse-biased above its breakdown voltage. When a photon generates an electron–hole pair, an avalanche is triggered and detected as a current pulse; after each detection, the avalanche is quenched, and the pixel is recharged before it can detect another photon, with typical dead times of 5–10 ns for the sensor used in this study [19], which was developed as part of a doctoral thesis [2] at the Institute of Microelectronics of Seville (CSIC-US). The specific device is a 64 × 64-pixel array with adaptive pixel sensitivity and an asynchronous readout [16]. Table 1 summarizes the sensor’s main specifications.

The array’s 3.5% fill factor (Table 1), corresponding to an effective photon-detection probability of about 2.6% over the full 24.5 μm × 24.5 μm pixel once the 75% peak PDP is accounted for, is lower than that of conventional image sensors but is typical of AER-based SPAD arrays: each pixel dedicates part of its area to in-pixel memory for state storage and to the ripple counter used in the on-chip AER address sequencer rather than to a TDC [16]. The device was also fabricated in a standard front-side-illuminated process rather than a backside-illuminated one, so this in-pixel circuitry sits directly on top of the photosensitive area and further reduces the light-collecting fraction of the pixel instead of being routed behind it as in a backside-illuminated design. In addition, each group of four neighboring SPADs (a 2 × 2 arrangement) shares a single deep n-well (DNW) cathode, a fabrication choice made because DNW design rules are otherwise highly restrictive and would further penalize the active area [2]; this shared-cathode arrangement, revisited in Section 4.4 in connection with the dark-count map, trades a small amount of electrical crosstalk between the four grouped pixels for an additional fill factor. Because the fill factor sets a hard ceiling on the fraction of incident photons that can reach an active detector, it directly limits sensitivity under photon-starved conditions. The consequence for intensity interferometry is quantitative and linear: the interferometer’s signal-to-noise ratio corresponds to ${\left(S/N\right)}_{s}=\alpha An_{\nu }\eta \;\Gamma \left(u,v\right)\;{\left(t\Delta f/2\right)}^{1/2}$, where α is the detector’s quantum efficiency; *A* is the collecting area; $n_{\nu }$ is the incident photon flux per unit time, area, and bandwidth; η is the instrumental efficiency; Γ(u,v) is the source’s degree of spatial coherence; *t* is the integration time; and Δf is the electronic bandwidth [20]. Because ${\left(S/N\right)}_{s}$ scales with α but only with $t^{1/2}$, matching the SNR of a detector with higher effective quantum efficiency requires integration times scaling as $1/\alpha^{2}$: with this sensor’s $\alpha_{\mathrm{eff}}\approx 2.6\%$ (corresponding to the fill factor times peak PDP) as the reference point, a detector reaching, e.g., four-times-higher effective quantum efficiency would need only about one sixteenth of the integration time for the same SNR. This is precisely the quantitative motivation—beyond the fill-factor discussion above on its own—for the higher-efficiency SPAD sensor mentioned below as the project’s next hardware generation (Section 4.4); the sensor’s previous astronomical validation (Section 2.2) was carried out on a bright star (Pollux, magnitude 1.1), so its performance under the much-fainter, background-limited conditions typical of intensity-interferometry targets remains to be characterized. Both limitations are consistent with the sensor’s origin as a LiDAR-optimized, front-side-illuminated device rather than one designed for astronomical photon budgets from the outset: given the sensitivity and readout results reported here, adapting this class of LiDAR-grade, event-driven SPAD technology to astronomical photon counting is a promising direction worth pursuing further, and our system should be interpreted as a proof of concept toward that goal rather than as a final, astronomy-optimized detector.

Most SPAD arrays developed for direct time-of-flight LiDAR and 3D imaging integrate a time-to-digital converter (TDC) at the pixel or column level and expose the resulting timestamps or histograms through a synchronous, clocked digital interface [4,6,8]. The device used in this work has a different, less common design: rather than an in-pixel TDC and a clocked readout, it communicates individual photon detection asynchronously and in an event-by-event manner through the Address-Event Representation (AER) protocol [13,16] on the basis of a request/acknowledge exchange that enables efficient, low-latency transfer without a global sampling clock. Each event carries only the pixel coordinates (X, Y). The protocol itself does not encode a timestamp, so any temporal information has to be supplied downstream by the acquisition electronics rather than by the sensor, as detailed in Section 3.2. This asynchronous protocol perfectly matches the operation of neuromorphic processing systems but, as will be shown later, it is also of interest for intensity interferometry.

In recent years, SPAD technology as a whole has undergone significant improvements—higher spatial resolution, integration of timing circuitry (TDCs), noise (DCR) reduction, and mitigation of parasitic effects such as crosstalk and afterpulsing [21,22,23,24,25]—which have driven its adoption across ToF LiDAR, 3D imaging, quantum communications, and biomedical imaging [5,26,27,28,29,30], consolidating its role as a mature technology in the industrial and commercial domain.

### 2.2. Intensity Interferometry in Astronomy

Intensity interferometry yields ultra-high angular resolution by correlating the intensity fluctuations detected simultaneously by two or more spatially separated telescopes [12]. Because it does not require preservation of the phase coherence of the wavefront, it is robust against atmospheric turbulence. Exploiting these correlations requires very precise temporal synchronization between nodes, typically achieved with GPS-disciplined clocks and protocols such as White Rabbit [31,32,33,34], which provides sub-nanosecond time transfer over Ethernet networks.

Despite the advantages SPAD arrays offer for this kind of application, their use in astronomical instrumentation remains very limited. The few existing studies in this line of research have demonstrated the feasibility of capturing fast astronomical events with SPAD arrays via multi-station intensity interferometers, analyzing photon statistics and temporal correlations between telescopes [10], and more recent work has extended the technique to kilometer-scale distances through active illumination, achieving resolutions fourteen times beyond the diffraction limit [9]. Illustrating the renewed momentum in the field, modern low-jitter time-tagging electronics and single-photon avalanche detectors have very recently enabled second-order photon-correlation detection with small, low-cost telescopes, recovering the expected squared visibility of Sirius at a 3.3 m baseline using a pair of 0.25 m telescopes [35]. In the context of this work, the SPAD sensor employed was previously validated at the Sierra Nevada Observatory (IAA-CSIC), where dark-count measurements and observations of the star Pollux (β Geminorum, magnitude 1.1) confirmed its ability to operate in a real astronomical environment. Moreover, a 64 × 64 SPAD array of this type was recently used to record the single-photon flux during the occultation of Betelgeuse by the asteroid (319) Leona, illustrating its ability to capture fast astronomical events [36].

Beyond SPAD-array-based approaches, other groups have developed dedicated readout electronics for photon-counting stellar intensity interferometry using different detector technologies. The electronics built for the stellar intensity interferometer of the ASTRI Mini-Array telescopes couple a compact, four-pixel silicon-photomultiplier (SiPM) array with discrete leading-edge discriminators and an FPGA backend, achieving a channel-to-channel timing skew of about 10 ps and a combined throughput of up to 80 Mcps across its four channels [37]. Similarly, an on-sky-demonstrated intensity interferometry instrument built around two hybrid photon detectors (HPDs) operated in single-photon-counting mode has achieved a coincidence-timing resolution of 42.4 ps (FWHM) at a per-detector count rate of ≈2 Meps, as validated through observations of the bright stars Vega, Altair, and Deneb made using the Calern Observatory’s 1.04 m C2PU telescope [38]. These systems illustrate a design point that is complementary to the one explored here: the prudence of using a small number of high-bandwidth, individually timestamped channels rather than a spatially resolved array. The 64 × 64 SPAD array and AER readout electronics developed in this work instead prioritize spatial multiplexing, with 4096 independently addressable pixels over a single acquisition bus, at the cost of not yet implementing per-event timestamping, which Section 3.2 and Section 4.4 identify as the main item to address before matching the temporal precision of dedicated timestamping electronics such as those cited above. Table 2 summarizes this comparison quantitatively; because the cited systems wire a handful of channels individually rather than multiplexing thousands of pixels over one shared bus, their much-higher aggregate rates and the per-pixel bus budget reported in Section 4.4 reflect different design points rather than a direct capability gap.

This study takes a step forward by exploring the integration of the sensor into a scalable acquisition architecture aimed at multi-telescope deployment within the LPQI project.

## 3. System Architecture and Implementation

**Figure 2:**
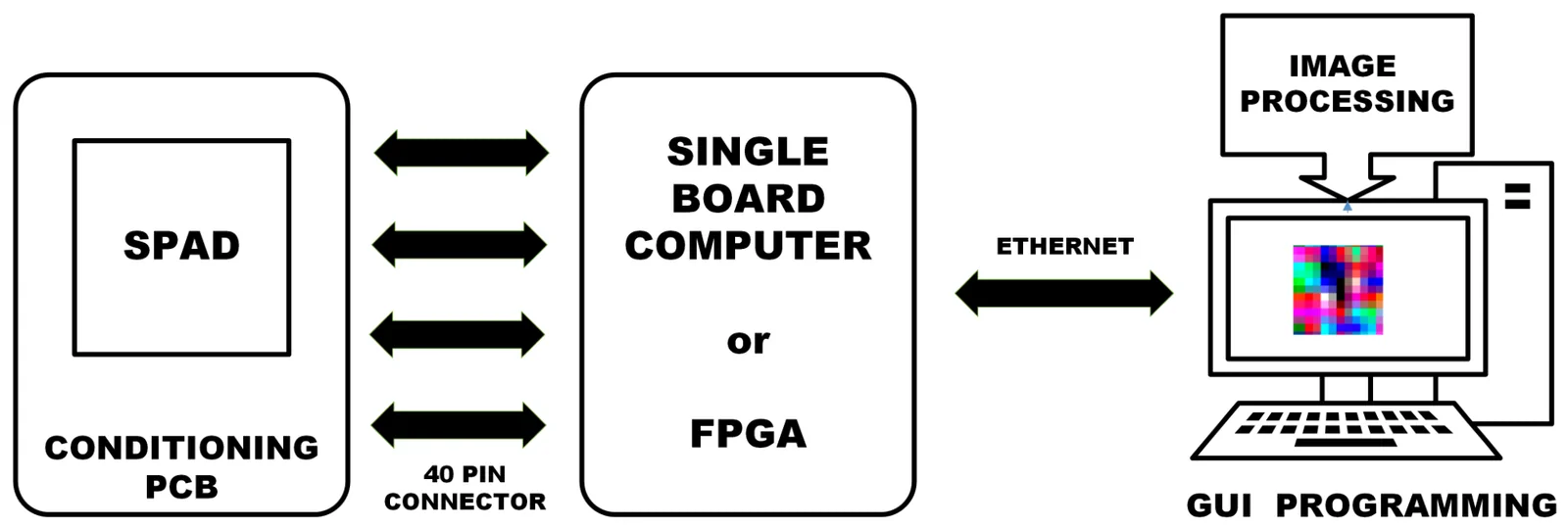
Block diagram of the system architecture. Through a conditioning PCB, the SPAD array connects to the processing platform (single-board computer or FPGA), which packages and streams the events to a host PC over the network for reconstruction, visualization, and storage.

To meet the high-speed single-photon acquisition requirements of intensity interferometry, a modular, scalable architecture was developed; a general block diagram of it is shown in Figure 2. The system is designed for real-time event detection, low-latency data management, and precise synchronization between distributed nodes. Given the complexity of the problem, we adopted a two-stage approach that separates flexible prototyping from high-performance processing, enabling iterative development and a progressive transition toward the final design to be installed in the telescopes at the Roque de los Muchachos. In the current version, the system does not implement timestamping; this functionality will be integrated as future work through the White Rabbit protocol, enabling precise temporal alignment between geographically distributed telescopes with a target resolution on the order of 500 ps, by combining a common White-Rabbit-disciplined clock and pulse-per-second (PPS) signal at each telescope node with a per-event time-to-digital converter (TDC) for sub-period interpolation, as detailed in Section 4.4.

### 3.1. First Stage: Raspberry Pi Prototyping

In the initial stage, a Raspberry Pi 5 development platform was used to drive the SPAD sensor over the AER protocol through GPIO, with sensor configuration handled over SPI, in order to validate the conditioning PCB and develop the acquisition and visualization software ahead of the FPGA implementation. Because the AER handshake was managed entirely via software, this stage restricted the capture rate to a few kHz, which is well below the sensor’s capacity, and was used only for functional validation; the same 40-pin conditioning PCB was reused in an unmodified form in the FPGA stage described next, and further architectural details on the Raspberry Pi prototyping stage are provided in Appendix A.

### 3.2. Second Stage: High-Speed FPGA Backend

**Figure 3:**
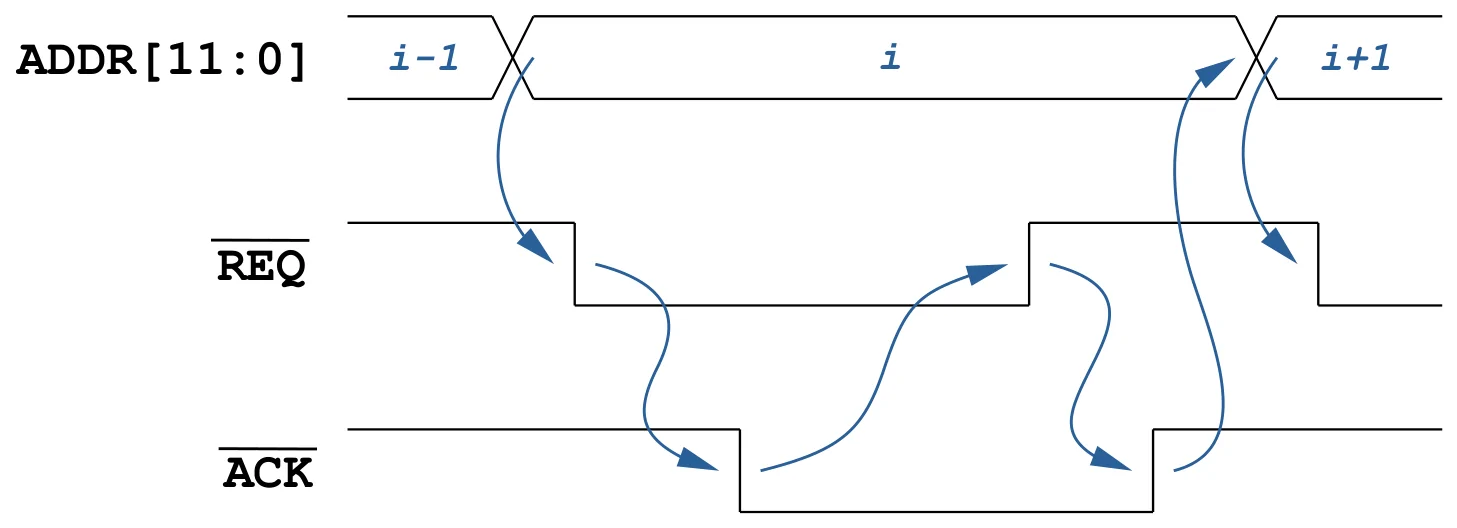
The four-phase AER handshake between the SPAD sensor and the FPGA AER module, shown for two consecutive events ($\bar{\mathrm{REQ}}$ and $\bar{\mathrm{ACK}}$ active low). The exchange is fully asynchronous and self-timed: there is no shared clock or fixed time grid, and the blue dashed arrows show how each transition directly triggers the next, whenever it occurs. Once the address for event *i* is placed on the bus, the sensor asserts $\bar{\mathrm{REQ}}$; the AER module then samples the address and asserts $\bar{\mathrm{ACK}}$ in response. Once the sensor observes $\bar{\mathrm{ACK}}$ low, it releases $\bar{\mathrm{REQ}}$, and the AER module releases $\bar{\mathrm{ACK}}$ once $\bar{\mathrm{REQ}}$ is observed to be high high again, returning the bus to the idle state and allowing the address for event i+1 to be placed.

The second stage features migration to an AMD Kria KR260 (Zynq UltraScale+ MPSoC) platform, which combines ARM Cortex-A53 processors with reconfigurable logic, in our case operating at 100 MHz. This architecture allows the AER handshake to be implemented entirely in hardware, removing the latency and jitter introduced by software. The few-kilohertz ceiling observed in the first stage (Section 3.1) stems from this software-driven handshake: each REQ/ACK cycle there requires a GPIO read/write pair mediated by the Python interpreter and the operating system’s scheduler, whose latency is dominant over the sensor’s own response time. Implementing the same handshake as dedicated logic clocked at 100 MHz, as performed in this second stage, removes the software-scheduling bottleneck entirely and lets the handshake run at the clock-cycle timescale; the ceiling on sustained throughput is then set instead by the sensor’s own AER arbitration (up to 40 Meps (Table 1)) and by how fast the FIFO can be drained toward DDR memory (Section 4.4). Section 4.4 further derives a tighter, handshake-specific ceiling from the per-transaction cycle count observed with the ILA. The handshake itself follows the conventional four-phase AER exchange, as illustrated in Figure 3: (i) the sensor places the pixel address on the 12-bit bus and, once it is stable, asserts $\bar{\mathrm{REQ}}$ (active low); (ii) the AER module samples the address and asserts $\bar{\mathrm{ACK}}$ (active low) once it is safely registered; (iii) the sensor observes $\bar{\mathrm{ACK}}$ low and releases both the address bus and $\bar{\mathrm{REQ}}$; and (iv) the AER module releases $\bar{\mathrm{ACK}}$, returning the bus to its idle (with both signals high) state and preparing it for the next event. Each phase is triggered directly by the previous one rather than by a shared clock edge, constituting an asynchronous, self-timed causal chain indicated by the diagonal arrows in Figure 3.

The SystemVerilog design includes the following main modules: the AER module, which captures the X/Y coordinates from the 12-bit bus, generates the acknowledge signal, formats each event into a 16-bit word (bits [5:0] = X coordinate, bits [13:8] = Y coordinate, 4 reserved bits), and asserts the TLAST signal every 4096 events to delimit DMA transfer blocks; DMA pipelines in Stream-to-Memory-Mapped (S2MM) mode for efficient transfer to the DDR memory of the processing system; and AXI4-Stream FIFO buffers to absorb event bursts and avoid data loss. This 16-bit word carries only spatial information: it does not include a time field, so the current implementation does not timestamp individual photon arrivals; this capacity is deferred to the White Rabbit integration discussed in Section 4.4 and Section 5. High-level control is performed through PYNQ, a Python-based interactive development environment running on the FPGA that configures the AXI peripherals, manages the DMA transfers, and visualizes the captured data. Migration to a bare-metal C/C++ implementation with AMD Vitis, thereby removing the overhead of the Python interpreter and reducing system latency, is planned as future work.

### 3.3. FPGA Implementation

**Figure 4:**
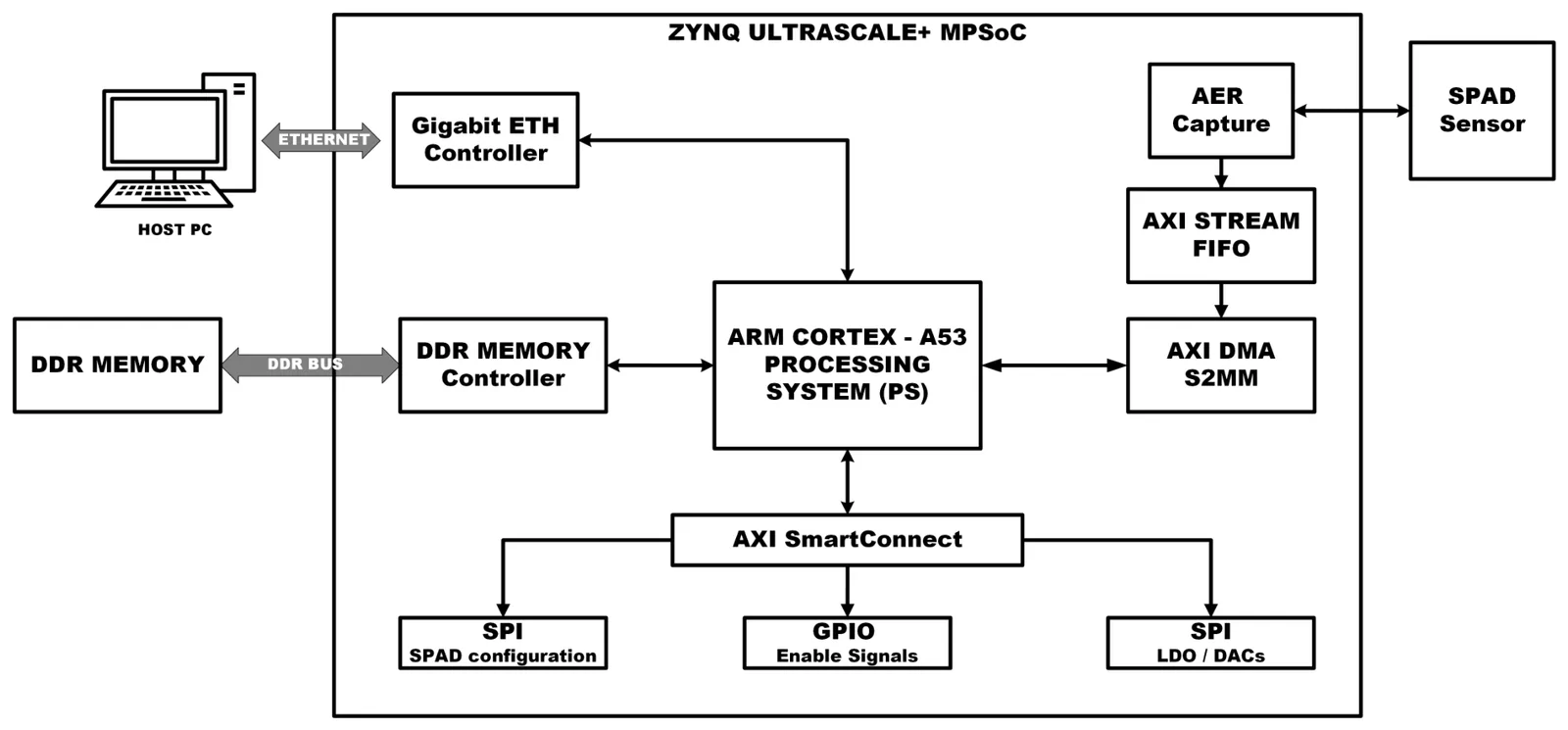
System block diagram on the Zynq UltraScale+ MPSoC chip, highlighting the physical separation between the Processing System (PS) and the Programmable Logic (PL). SPAD events are captured by AER logic in the PL and streamed via AXI DMA directly to the PS DDR controller, where they are stored in external DDR memory. The PS processes the data and transmits them to a Host PC via Gigabit Ethernet while managing sensor parameters via SPI and GPIO peripherals connected through AXI SmartConnect.

The complete FPGA implementation integrates the AER acquisition pipeline described in Section 3.2 with the memory and communication infrastructure of the Zynq UltraScale+ MPSoC, as shown schematically in Figure 4. The AER, DMA, and FIFO modules were each independently validated via simulation before joint integration. The SPAD sensor receives a 20 MHz external clock generated by a crystal oscillator on the conditioning PCB, ensuring that there is a stable temporal reference independent of the acquisition platform and decoupled from the 100 MHz clock domain of the AER/AXI pipeline. On the processing-system side, the ARM Cortex-A53 cores configure the AXI peripherals and manage the DMA transfers through PYNQ, while the SPI and GPIO peripherals used for sensor configuration are connected through AXI SmartConnect, keeping the configuration path physically and functionally separate from the high-rate event-acquisition path.

### 3.4. Conditioning PCB

**Figure 5:**
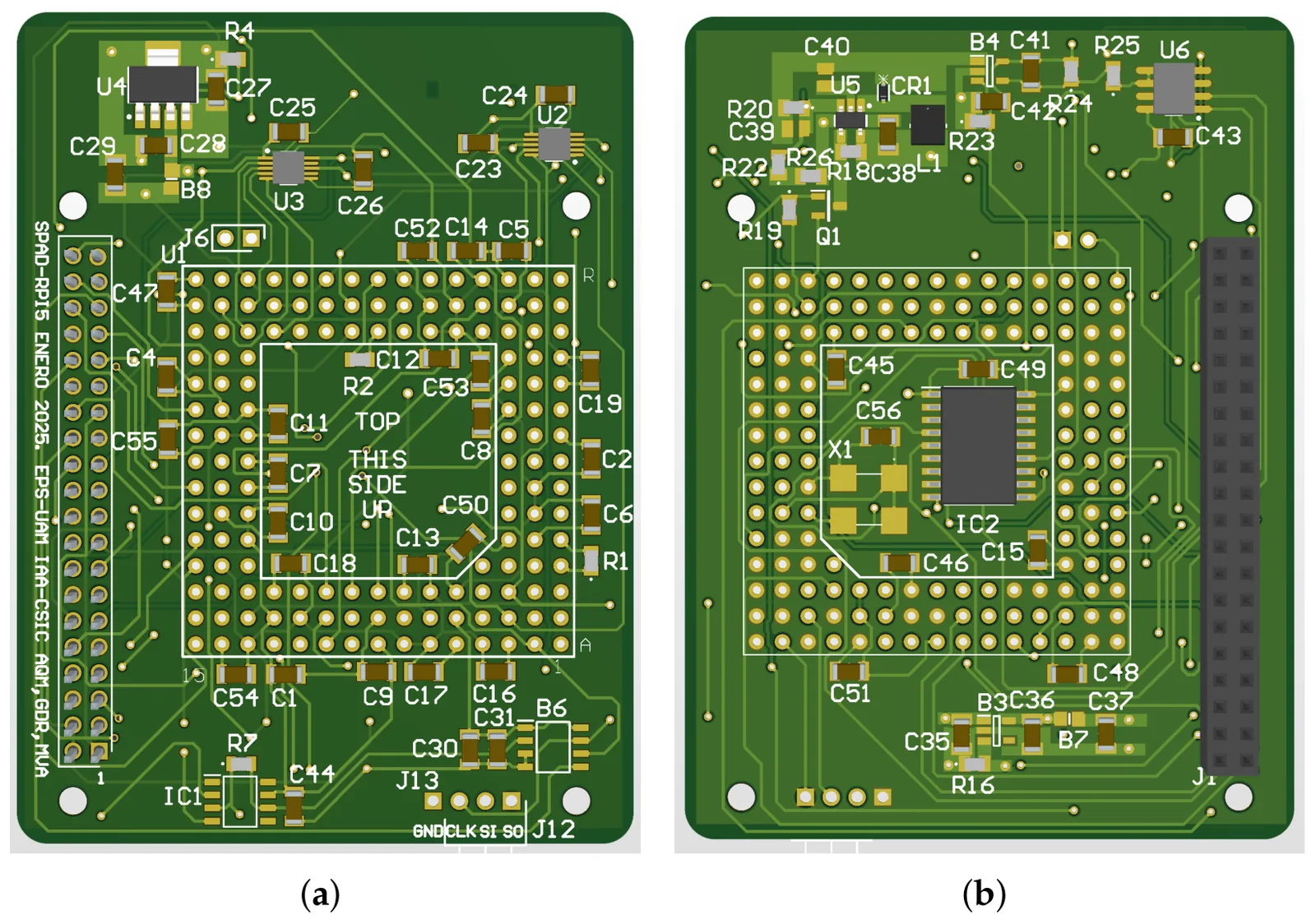
3D model of the four-layer conditioning PCB: (**a**) top layer and (**b**) bottom layer.

Figure 5 shows the conditioning PCB, designed in four layers: two outer signal layers and two inner layers dedicated to the power plane and ground plane. This configuration allows clean routing of the high-frequency AER bus signals and reduces the electrical noise induced by the DC-DC converters. Traces were routed at 45° to avoid impedance discontinuities and minimize electromagnetic interference. Power management includes the LP38693 regulator (3.3 V) for the digital logic and the sensor, the TPS7A05 regulator (1.2 V) for the SPAD core, and the LMR64010 boost converter together with the NCP730 LDO to generate the SPAD bias voltage, which is adjustable above 18 V via an MCP41010 digital potentiometer. The MCP23S09 GPIO expander, controlled over SPI, provides the additional pins required for full sensor control.

### 3.5. Timing Closure and Resource Utilization

Timing analysis of the design confirmed the Worst Negative Slack (WNS) was 2.219 ns on the 100 MHz clock, equivalent to a maximum achievable frequency of approximately 128 MHz, leaving sufficient margin for future extensions of the design. Table 3 summarizes the resource utilization of the implementation on the AMD Kria KR260. The design makes moderate use of the programmable logic, with 9.89% of LUTs and 6.92% of flip-flops, while BRAM utilization is higher (79.51%), mainly because of the FIFO buffers of the AER acquisition pipeline; this leaves limited headroom to deepen those buffers for absorbing longer high-rate bursts, so scaling to sustained higher event rates would likely require a design that manages BRAM more sparingly, such as narrower per-event FIFO words or streaming events directly toward DDR memory. The total on-chip power dissipation is 2.839 W, a relevant parameter for the future deployment of the system in the telescopes, where the electronics—particularly the FPGA, which will be placed in a vacuum chamber—will have to operate under more-demanding thermal-dissipation conditions.

As a future extension, we foresee the integration of Aurora 64B/66B links for inter-FPGA communication and the incorporation of the White Rabbit protocol for sub-nanosecond temporal synchronization.

## 4. Experimental Validation

### 4.1. Optical Bench

We carried out validation on a laboratory optical bench, rather than directly on sky, in order to characterize the acquisition electronics, spatial localization, dynamic range, and noise under fully controlled and repeatable illumination conditions before committing to telescope integration. This process decoupled the performance of the readout chain from the additional variables introduced by atmospheric seeing, telescope optics, and night-time scheduling constraints at the observatory and allows the iris diaphragm aperture to be swept in a controlled, repeatable manner as a proxy for varying incident photon flux, playing a role broadly analogous to observing targets of different apparent brightness. On-sky validation of the full acquisition chain, once integrated with the LPQI telescopes, is planned as a subsequent step (Section 5).

**Figure 6:**
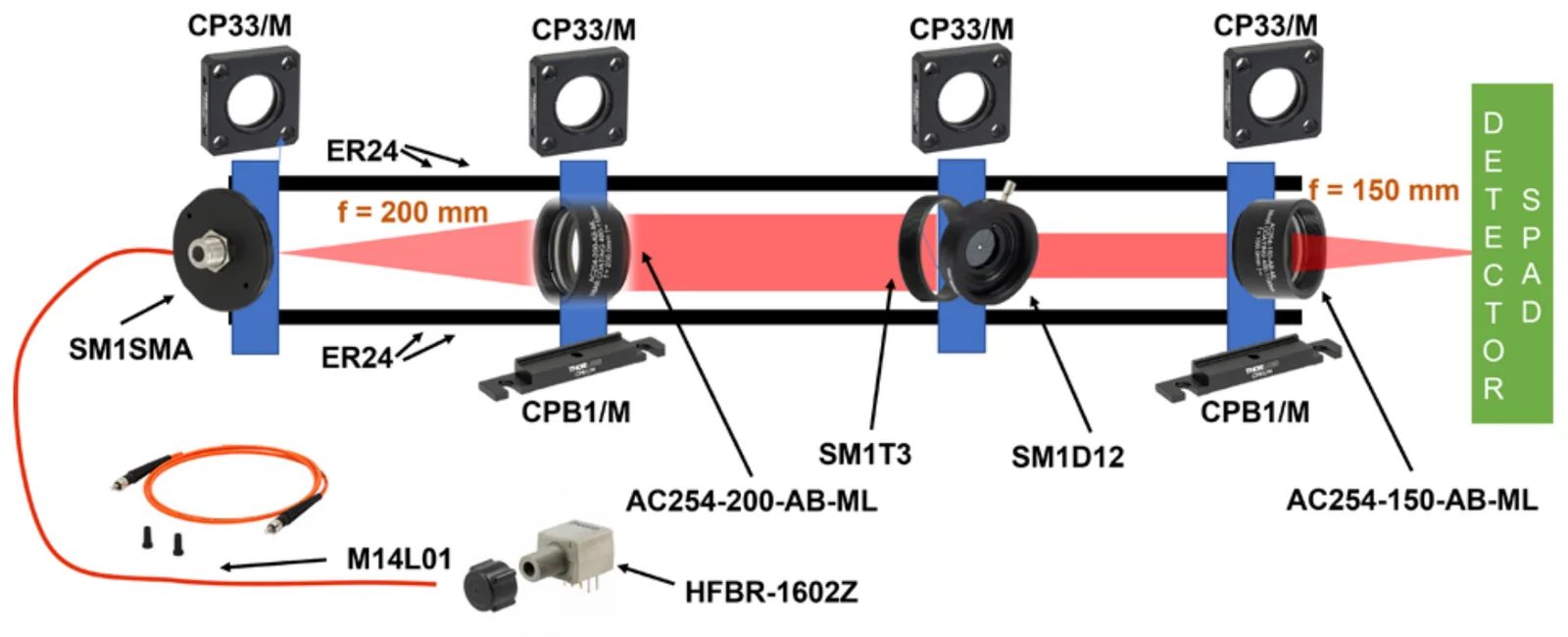
Optical bench design for validation: collimator, achromatic lenses, iris diaphragm, fine-adjustment fiber mount, and flip mirror for alignment.

Experimental validation was carried out under laboratory conditions on an optical bench with fiber-coupled sources and controlled illumination modulation, as shown in Figure 6. The bench includes a fiber collimator to parallelize the rays from the source, achromatic doublet lenses, an adjustable iris diaphragm, a fine-adjustment mount that aims the optical fiber at the center of the detector, and a flip mirror for prior alignment through an eyepiece; all optical and optomechanical components on the bench are commercial off-the-shelf parts from Thorlabs, Inc. (Newton, NJ, USA), listed in Table 4. During the Raspberry Pi 5 prototyping stage, an issue was identified in the SPI-based configuration interface between the conditioning PCB and the SPAD sensor, which initially prevented the acquisition of valid data. The issue was diagnosed and corrected through a revised PCB design, which also motivated the transition to the FPGA stage. Three verification tests of the complete system were performed: event acquisition under low illumination (1 mm iris aperture), event acquisition under high illumination (12 mm iris aperture), and dark-count-rate (DCR) characterization in the absence of illumination at different temperatures.

**Figure 7:**
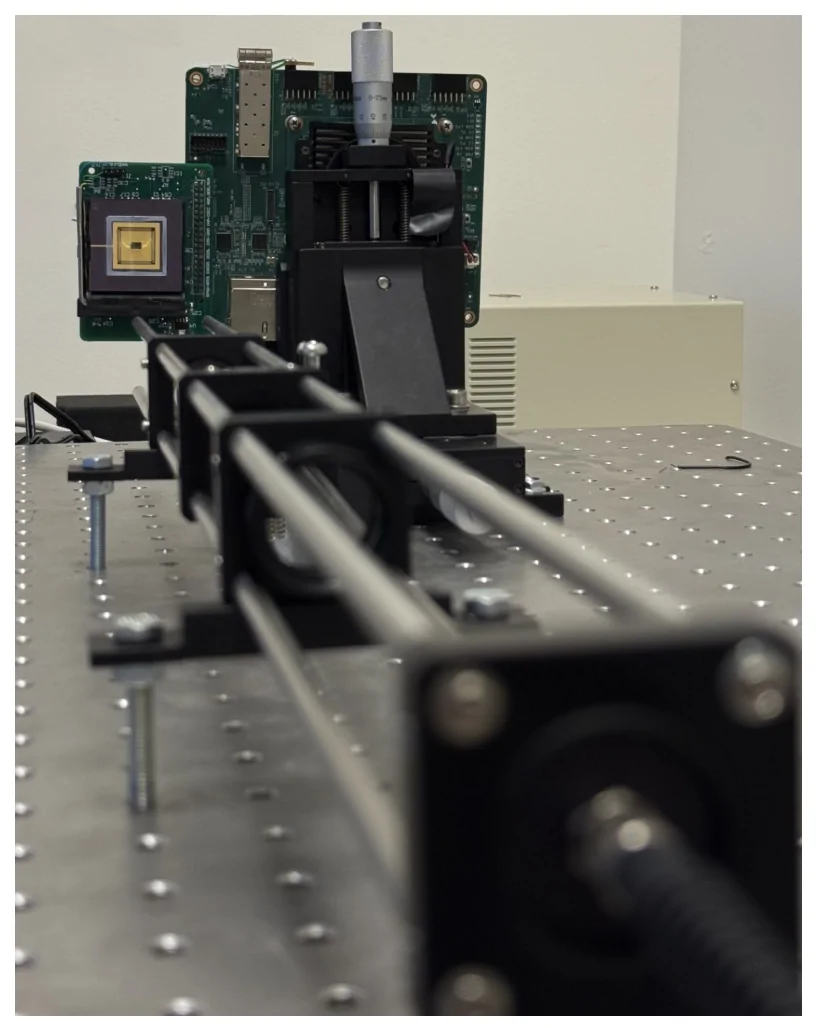
Physical implementation of the optical bench setup: the 64 × 64 SPAD array and conditioning PCB connected to the AMD Kria KR260 FPGA board, aligned with the collimated illumination path on the optical rail.

Figure 7 shows the corresponding physical implementation, with the SPAD sensor and conditioning PCB (Section 3.4) mounted at the detector end of the bench, in line with the collimated illumination path, and connected to the AMD Kria KR260 FPGA board.

### 4.2. AER Protocol Validation with the ILA

Prior to the optical tests, we evaluated whether the acquisition system was operating correctly via the Integrated Logic Analyzer (ILA) core embedded in the FPGA, which captures internal signals at clock-cycle resolution without interfering with system operation. The traces confirmed that the REQ/ACK handshake between the sensor and the FPGA was correct, the capture of the X/Y coordinates during each event was stable, and the transmission over the AXI-Stream channel in 16-bit packets (bits [5:0] = X coordinate, bits [13:8] = Y coordinate) was correct. The TLAST signal was asserted correctly every 4096 events, marking the block boundaries for the DMA transfer. The traces further showed that completing one full REQ/ACK handshake and event capture, from the sensor’s initial address-and-$\bar{\mathrm{REQ}}$ assertion (Figure 3) to the AER module’s release of $\bar{\mathrm{ACK}}$ and return to the idle state, takes approximately 15 clock cycles at the 100 MHz system clock. Section 4.4 derives the corresponding theoretical throughput ceiling of the current handshake implementation from this figure. We also observed that at certain instants, the FIFO output TVALID signal dropped to zero, indicating potential bottlenecks in draining the buffer toward the DMA under bursts of high activity. Section 4.4 discusses how this observation relates to the event counts reported in Section 4.3.

### 4.3. Optical Results

**Figure 8:**
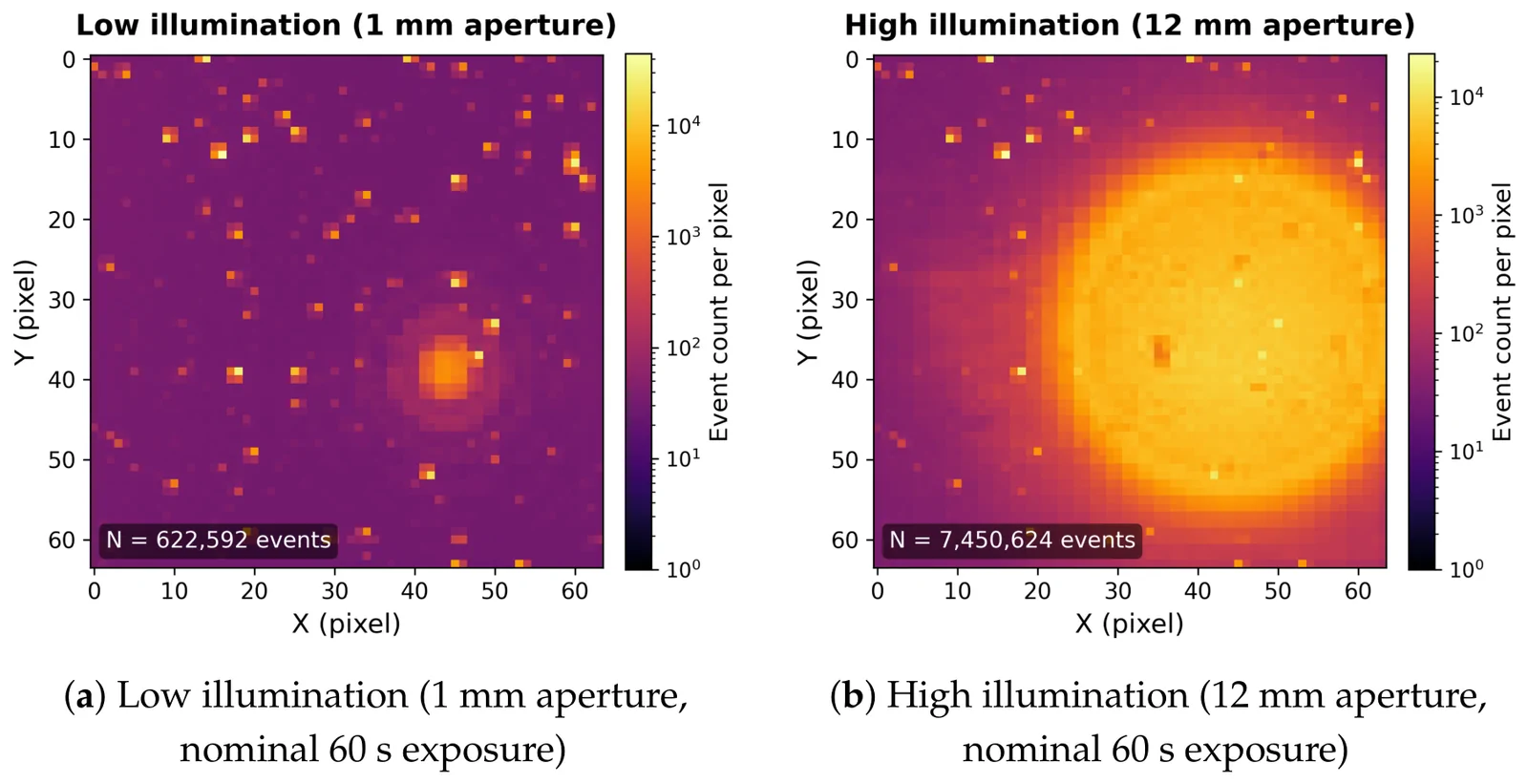
Optical validation results (nominal 60 s exposure in both cases—see Section 4.3 for the DCR exposure durations): (**a**) accumulated AER events under low illumination, showing a localized photon cluster, and (**b**) response under high illumination, delimiting the illuminated area.

Figure 8a shows the accumulation of AER events under low illumination (1 mm aperture, approximately 60 s exposure). A localized cluster of events associated with the faint source is shown, while the rest of the array shows scattered activity due to background noise. Despite not being carried out in an entirely dark chamber, the system spatially localizes the photonic signal clearly, demonstrating the sensitivity of the sensor and correct AER decoding by the FPGA. Figure 8b shows the response under high illumination (12 mm aperture, approximately 60 s exposure): the central region presents a saturated pattern with high per-pixel counts, precisely delimiting the illuminated area; the presence of ambient light does not prevent identification of the source, evidencing the dynamic range of the sensor. The raw AER event streams for both captures were logged and decoded offline, yielding total counts of 622,592 events under low illumination and 7,450,624 events under high illumination; division by the approximately 60 s exposure common to both captures yields measured aggregate event rates of ≈10.4 keps and ≈124.2 keps, respectively, which are comfortably below the sensor’s 40 Meps arbitration ceiling and the per-pixel bus budget discussed in Section 4.4. As exposure duration was not logged with sub-second precision, these figures should be interpreted as being approximate, order-of-magnitude throughput measurements rather than calibrated timing references; a systematic throughput sweep under controlled, precisely timed background levels remains one of the open items identified in Section 4.4.

**Figure 9:**
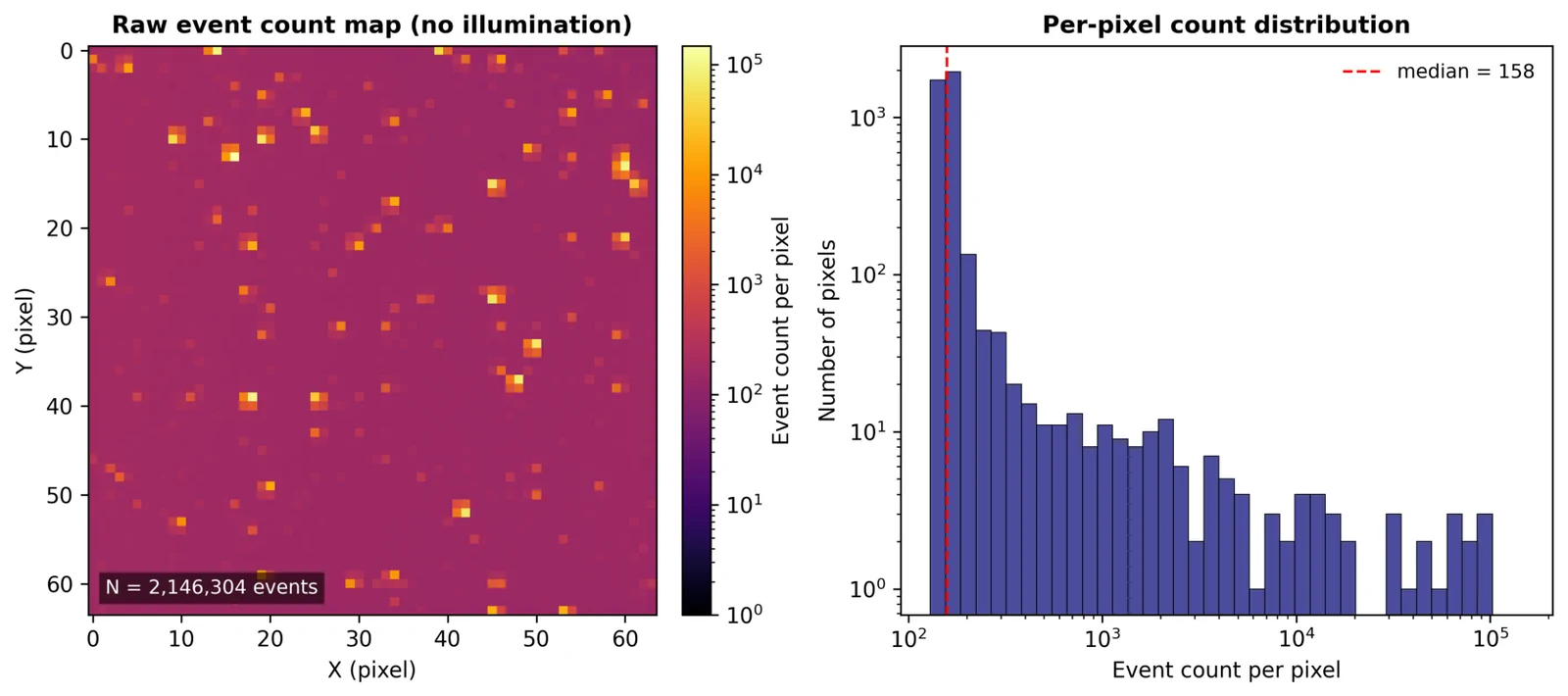
Dark-count-rate (DCR) characterization of the 64 × 64 array via a raw AER event capture at 27.8 °C, decoded offline (2,146,304 events, with approximately 90 s exposure, recalled rather than logged with sub-second precision). Left: Per-pixel raw event-count map on a logarithmic color scale, where isolated hot pixels stand out against a background mostly below a few Hz. Right: The corresponding per-pixel count distribution, with a median of 158 counts per pixel (≈1.76 Hz), consistent with the independently measured 1.68 Hz reported for the same nominal temperature over a precisely logged 59.9 s exposure (Section 4.3).

The dark-count rate (DCR) of the array was characterized at two temperatures via precisely logged captures, namely, a median of 1.68 Hz at 27.8 °C over 59.9 s of exposure and a median of 3.61 Hz at 34.5 °C over 59.5 s, evidencing the thermal dependence of the DCR. In both cases, we identified isolated hot pixels with rates well above the median, which had to be excluded during the correlation analysis. Figure 9 shows an independent repeat dark capture at the same nominal 27.8 °C, with its raw per-pixel event counts logged and decoded offline (2,146,304 events in total; median: 158 counts per pixel) over an approximately 90 s exposure; as with the exposure durations in Section 4.3, this duration was recalled rather than logged with sub-second precision. This repeat capture gives a median DCR of ≈1.76 Hz, within ≈5% of the 1.68 Hz reported above, supporting the repeatability of the measurement as an approximate consistency check rather than a calibrated repeat. Having the raw per-pixel counts for this capture also allows the shape of the DCR distribution to be quantified directly: 97.2% of pixels lie below 10 Hz, and 99.2% lie below 100 Hz, while a long tail of 31 pixels (≈0.8% of the array) exceeds 100 Hz, with the single hottest pixel reaching ≈1.6 kHz; this heavy-tailed shape is consistent with the sparse, isolated hot pixels visible in Figure 9 and reinforces the rationale for excluding them individually rather than applying a single global correction.

**Figure 10:**
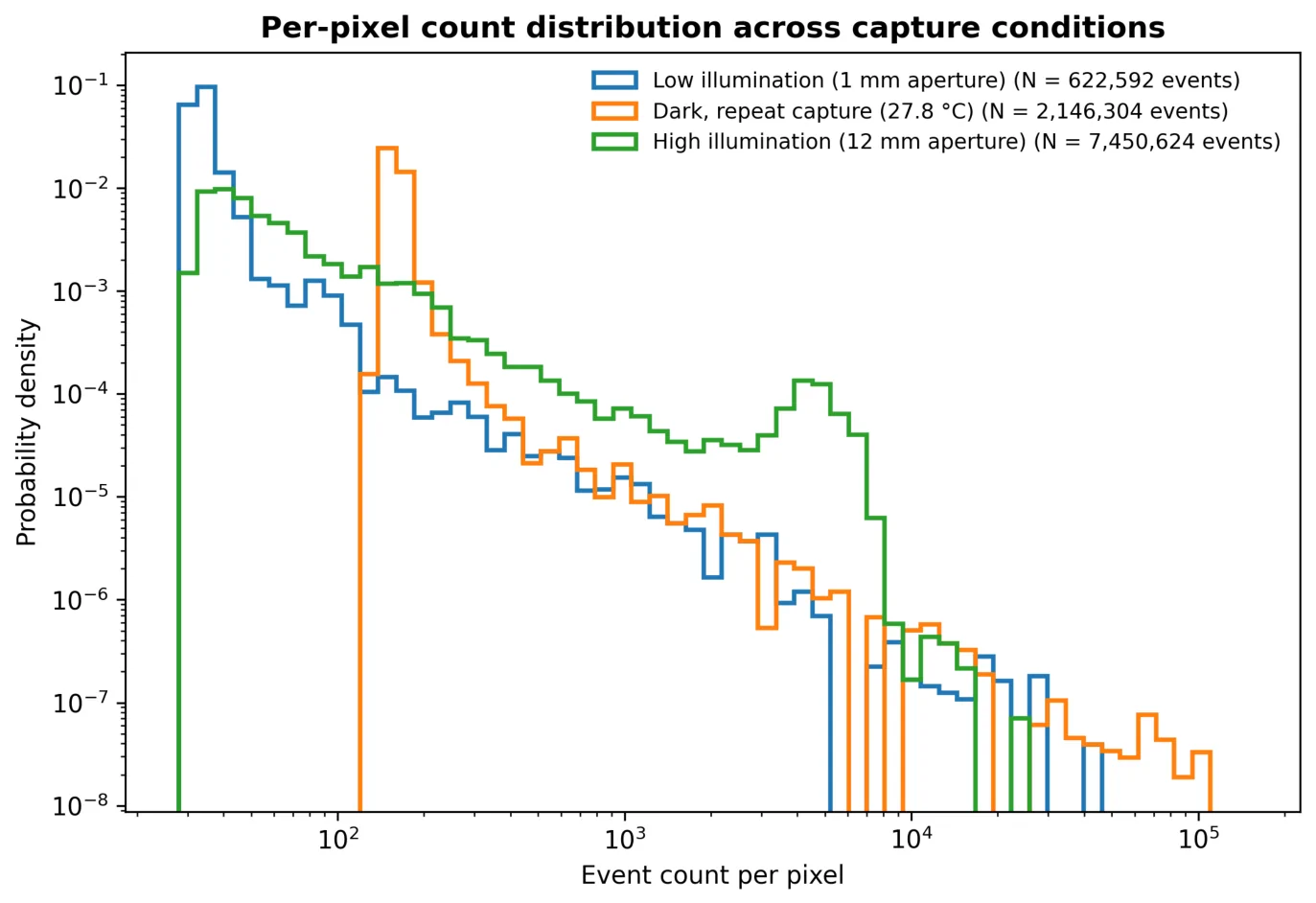
Per-pixel count distributions of the three raw AER captures analyzed in this work: low illumination (1 mm aperture, 622,592 events), the dark repeat capture at 27.8 °C (2,146,304 events), and high illumination (12 mm aperture, 7,450,624 events). The dark and low-illumination distributions overlap closely at low counts; the high-illumination capture shows a distinct second mode near $4–5\times 10^{3}$ counts per pixel, corresponding to the illuminated spot’s photon signal.

Figure 10 compares the per-pixel count distributions of all three raw AER captures decoded in this work: the low- and high-illumination captures in Figure 8 and the dark repeat capture in Figure 9. The low-illumination and dark distributions overlap closely at low counts, as expected given the modest signal added by the 1 mm aperture, while the high-illumination distribution departs clearly from both, showing an additional bump near $4–5\times 10^{3}$ counts per pixel; this second mode is the illuminated spot’s photon signal superimposed on the same underlying background distribution, and its separation from the low-count peak is itself visual confirmation that the acquisition chain distinguishes the two regimes correctly.

### 4.4. Discussion

Taken together, these results demonstrate that a modular, FPGA-based AER acquisition architecture can be built around a LiDAR-conceived, event-driven SPAD array to detect and spatially localize photon events, directly addressing the architectural gap identified in Section 1, at a measured throughput of up to ≈124 keps under the highest illumination condition tested (Section 4.3). Reaching and sustaining the higher, multi-hundred-keps-to-Meps rates that bright-source intensity-interferometry targets would ultimately require has not yet been tested under a controlled, systematic sweep and remains assessed only analytically below. The results confirm the feasibility of using the acquisition electronics for detecting and localizing photon-based events under different illumination conditions. The thermal dependence of the DCR, which doubles for a temperature increase of about 7 °C, reinforces the need to incorporate active cooling, such as a Peltier stage, in final telescope deployment. A further observation regarding the dark-count map (Figure 9) is that hot pixels do not appear fully isolated: neighboring pixels sharing a fabrication-level structure can show an elevated dark-count rate when one of them is a hot pixel. The four pixels within each affected group are independent SPAD detectors, each with its own avalanche junction and in-pixel ripple counter and SRAM state memory; the coupling arises not from shared digital AER logic but from the shared deep n-well (DNW) cathode across each 2 × 2 group of four pixels introduced in Section 2.1, which was adopted to relax otherwise-restrictive DNW design rules and improve the fill factor. This shared-cathode arrangement is a known source of electrical and, to a lesser extent, optical crosstalk between the four pixels of a group: an equivalent 2 × 2 pattern in the dark-count map, together with a quantitative crosstalk characterization (up to ≈1.9% between shared-cathode neighbors, and below 0.05% otherwise), has been reported for this sensor architecture [2]. This shared-cathode grouping was independently confirmed directly on our own array: scanning the four possible 2 × 2 phase offsets against the per-pixel count maps from multiple raw captures analyzed in this work (Section 4.3), including both illuminated and dark conditions, reveals a single consistent registration (pixels grouped in phase with a one-row, one-column offset from the array origin) is the correct one, with a spatial correlation between grouped pixels of ρ≈0.81 (Spearman) and r≈0.69 (Pearson), against essentially zero correlation for a randomly shuffled null baseline. This confirms that the grouping pattern itself, not only its existence, matches the shared-cathode fabrication layout. A full quantitative characterization of the crosstalk magnitude in our own array, comparable to the percentage figures cited above, is left for future work. In the meantime, grouped hot pixels are excluded together during the correlation analysis to avoid biasing the noise budget. This observation, together with the fill-factor and throughput limitations discussed above, has directly informed an ongoing effort, beyond the scope of this paper, to design a new SPAD sensor and its matching control electronics specifically for the LPQI project, so the characterization reported here is already being fed forward into the project’s next hardware generation. The current main limitation is the absence of precision timestamping, which is required for the temporal correlation between telescopes in intensity interferometry. This functionality is planned as future work through the integration of the White Rabbit protocol: each telescope node would receive from White Rabbit a common, sub-nanosecond-synchronized clock together with a pulse-per-second (PPS) signal, and a time-to-digital converter (TDC) added to the AER module would timestamp each event locally with respect to the nearest PPS edge; combining the White Rabbit-disciplined absolute time base with the TDC’s sub-period interpolation would let events captured at geographically separated telescopes be placed on a common time reference for later correlation, with the goal of reaching resolutions of on the order of 500 ps.

The TDC itself remains a design choice for future work, but the two stages it would combine are both established techniques rather than open research questions. The coarse stage, yielding the timestamp’s integer component, would be a free-running counter clocked from the WR-recovered reference (or from the existing 100 MHz AER/AXI-Stream domain of Section 3.2, kept aligned to WR via the PPS edge); at the 500 ps scale, this stage carries no meaningful resolution limitations of its own. The fine stage, handling the sub-period interpolation within one coarse tick, is where the achievable resolution is actually set: the natural implementation on this platform is a tapped-delay-line (TDL) time-to-digital converter built directly from the FPGA’s own carry-chain primitives, in which the event edge propagates through a chain of fast logic elements and, at the next clock edge, the pattern of taps it has already reached is latched as a thermometer code; after bin-by-bin calibration, this code provides the sub-period offset. This is a mature technique specifically for Xilinx UltraScale and UltraScale+ fabrics, which is exactly what the Zynq UltraScale+ MPSoC on the Kria KR260 used in this work provides: a TDL-based TDC implemented on a 16 nm UltraScale+ device, the same programmable-logic family as this work’s second stage, has recently demonstrated a resolution of 1.15 ps and an RMS single-shot precision of 3.38 ps using a Bin Sequence Calibration procedure that combines partial-order reconstruction with iterative time-bin interleaving on code-density-test data to correct for the differential and integral nonlinearity inherent to the raw, uncalibrated tap delays [39]; on the closely related 20 nm UltraScale generation, a TDL TDC calibrated with a simpler bin-by-bin, code-density procedure exhibited a 2.64 ps RMS single-shot precision for its highest-precision configuration, at a cost of 883 configurable logic blocks (CLBs) and 126 kb of BRAM, alongside a lower-area variant reaching a 3.75 ps RMS precision in only 259 CLBs and 72 kb of BRAM [40]. Both results indicate that sub 10 ps precision in the fine stage, roughly two orders of magnitude below the ≈500 ps target, is routinely achievable on exactly this class of FPGA fabric, provided a calibration procedure built on code-density-test data, of the kind these two studies rely on, is carried out. Because such calibration compensates for process, voltage, and temperature variation rather than being a one-time factory step, it would need to be repeated periodically once the TDC is in operation. The BRAM cost reported for the higher-precision configurations is a genuine constraint here rather than an abstract one since BRAM is already the most heavily used resource in the current implementation (79.51%, as noted in Section 3.5); fitting a TDC alongside the existing AER acquisition pipeline would likely favor a lower-area TDL configuration or one that offloads calibration data to DDR memory rather than storing it entirely in BRAM.

Combined with the ≈10–20 ps WR synchronization jitter and the ≈81–96 ps end-to-end uncertainty already demonstrated with a full WR-plus-FPGA-TDC chain on a previous-generation Zynq device [34] and with the picosecond-level time deviation reported for a free-space WR link over 100 m [32], this comparison suggests that reaching the ≈500 ps target is governed less by the FPGA TDC’s own quantization, which the results above show can be pushed well into the low-picosecond range, than by the WR link’s synchronization jitter and by careful PCB-level clock distribution—in particular, matching cable and trace lengths for the PPS signal, and keeping the clock-domain crossing between the WR-recovered clock and the existing 100 MHz AER/AXI-Stream domain from adding uncertainty of its own. Such crossing does not exist in the current design, since the SPAD sensor’s 20 MHz clock and the 100 MHz AER/AXI domain are already independent (Section 3.2) and would need to be reconciled once WR-based timestamping is introduced. None of the figures cited in this paragraph were measured on the system presented here, and the specific TDC implementation, its calibration procedure, and the clock-domain-crossing scheme all remain to be designed and characterized; they are cited as evidence—from directly comparable or identical FPGA fabric—that the 500 ps target carries a substantial engineering margin, not as a result already achieved in this work.

On the throughput side, the 40 Meps figure in Table 1 is the sensor’s own on-chip AER arbitration ceiling, not a measured bound on the readout chain implemented here. Averaged uniformly over the 4096 pixels of the array, it corresponds to ≈9.8 kevents/s per pixel. A tighter, implementation-specific ceiling follows directly from the ILA traces (Section 4.2): completing one REQ/ACK handshake and event capture takes approximately 15 clock cycles at the 100 MHz system clock, so the current AER-to-AXI-Stream capture logic itself imposes a theoretical maximum sustained rate of 100MHz/15≈6.667 Meps aggregate, or ≈1.63 keps per pixel averaged uniformly over the array—a figure tighter than, and therefore superseding, the sensor’s own ceiling as the binding constraint on this specific implementation. As with the sensor’s figure, this remains a theoretical bound derived from the observed per-transaction cycle count rather than a measured sustained-throughput limit since it has not been validated under a saturating, back-to-back event stream. A background rate above this tighter level at any single pixel would be expected to saturate the handshake logic before the sensor’s own arbitration ceiling is reached, and the current design does not implement per-event loss or overflow accounting, so such saturation would not be reported explicitly. In particular, the REQ/ACK handshake is completed independently of whether the event is actually queued into the AXI-Stream pipeline, so an event arriving while a previous one is still awaiting acceptance downstream is acknowledged by the sensor but silently dropped before reaching the DMA, with no indication at either end; adding per-event overflow/drop accounting, as noted below, would also close this gap. The intermittent TVALID deassertion observed with the ILA during bursts of high activity (Section 4.2) is a symptom of this same mechanism. At the ≈124 keps measured under the highest illumination condition tested (Section 4.3), averaging to a per-pixel rate of ≈30 events/s across the array, this remains more than an order of magnitude below the tighter ≈1.63 kevents/s per-pixel budget set by the current handshake implementation, so silent loss is not expected to be significant under the specific conditions validated here; however, without a per-event loss counter, this expectation cannot be verified directly, and the reported raw event counts should accordingly be interpreted as what was captured downstream of the AER module rather than as a value independently confirmed to include every event the sensor produced. This is a practical constraint for deployment under a bright-sky background that is not yet characterized experimentally in this work: the event rates reported in Section 4.3, up to ≈124 keps, reflect the two fixed laboratory conditions used for validation, not a systematic sweep against a stressed, uniform background nor a precisely timed measurement. Quantifying the sustained throughput and loss behavior of the full readout chain under high background illumination, adding per-event overflow/drop counters to the FPGA design, and running a systematic, precisely timed throughput sweep beyond the two illumination conditions reported here remain open goals for the next experimental campaign. Likewise, beyond the clock-cycle-resolution functional and timing checks performed with the ILA (Section 4.2), a dedicated characterization of the end-to-end latency and bit-error rate of the AER-to-DMA pipeline under sustained high-rate operation was not carried out in this work, and the DCR characterization reported in Section 4.3 corresponds to a single ≈60 s capture at each of two temperatures rather than a multi-session repeatability study. Both are identified here as open items for future experimental campaigns.

Although full instrument design for the LPQI telescopes is beyond the scope of this work, first-order estimates of the angular resolution and expected sky-background rate can help assess the array’s suitability for the LPQI–Pathfinder pair. LPQI will measure single-photon correlations with angular resolutions down to 50 μas—approximately 1000 times sharper than those of the Hubble and James Webb space telescopes—ushering in a new era of high-resolution astrophysics. This breakthrough will open new avenues for exploring the nature of black-hole accretion disks, the structures of neutron stars, the dynamics of cosmic expansion from local supernovae, and the origins of ultra-fast transients. The LPQI–Pathfinder, using the NOT and TNG separated by a baseline B=550 m, will provide an angular resolution of θ∼λ/B≈150μas at λ=400 nm, the characteristic resolution of an intensity interferometer at that baseline and wavelength. Assuming the detector is placed directly at the Cassegrain (NOT, 2.56 m, *f*/11 [41]) or Nasmyth (TNG, 3.58 m, 38.5 m focal length [42]) focus, without additional relay optics, the 24.5 μm pixel pitch (Table 1) corresponds to a plate scale of about 0.18 arcsec/pixel at NOT and 0.13 arcsec/pixel at TNG, giving a field of view of roughly 11.5 arcsec and 8.4 arcsec, respectively, across the full 64 × 64 array, well matched to a point-source target under typical seeing at the site. These figures follow directly from the telescopes’ own explicitly stated focal lengths, 28.16 m and 38.5 m, respectively, rather than the site’s rounded *f*/11 label for TNG, whose corresponding ratio is closer to 10.75. Using the Roque de los Muchachos dark-time sky brightness of 21.70 mag/arcsec^2^ in the *V* band [43], the incident sky-background rate per pixel, before detector PDP and instrumental throughput, follows from

(1) $$N_{\mathrm{sky}}=F_{0}\;10^{-\mu /2.5}\;p^{2}\;A_{\mathrm{tel}}\;\Delta \lambda ,$$

where $F_{0}\approx 996$ photons s^−1^ cm^−2^ Å^−1^ is the *V*-band zero-point photon flux for an $m_{V}=0$ star obtained by converting the energy-flux zero point tabulated for this band ($f_{\lambda }=363.1\times 10^{-11}$ erg s^−1^ cm^−2^ Å^−1^ at the effective wavelength $\lambda_{\mathrm{eff}}=5450$ Å [44]) into photon units; μ is the sky surface brightness in mag/arcsec^2^; *p* is the plate scale in arcsec/pixel (so $p^{2}$ is the solid angle subtended by one pixel); $A_{\mathrm{tel}}$ is the telescope’s collecting area; and Δλ≈890 Å is a representative width for the Johnson *V* bandpass, adequate for the first-order estimate sought here. Evaluating Equation (1) for the NOT and TNG apertures yields only ≈3 photons/s per pixel at both telescopes; because the plate scale scales approximately as 1/D at a roughly fixed focal ratio, this figure stays close to the same order of magnitude for both apertures, which are operated near *f*/11 (NOT) and *f*/10.75 (TNG), as is the case for the LPQI–Pathfinder pair. This incident rate is of the same order as the already-measured dark-count rate (Section 4.3) and more than two orders of magnitude below the tighter ≈1.63 kevents/s per-pixel budget discussed above, so ambient sky background at this dark site is not expected to threaten bus saturation on its own; the relevant throughput constraint instead applies to the flux from the target star itself under bright-source conditions. This estimate assumes direct use at the telescope’s natural focus; if the final design couples the sensor through a focal reducer, fiber feed, or other relay optics, both the plate scale and the background rate would change accordingly and should be revisited once that design is fixed.

The captures from the Vivado ILA also localized the current performance bottleneck not in event capture but in draining the FIFO buffer toward memory, which is the natural target for optimization as the event rate increases. Finally, while the KR260, with its full processing system, is convenient for development, it is not optimal in power for the final deployment; a programmable-logic-centric device would be more appropriate, and its higher thermal demands inside the vacuum chamber must be considered.

## 5. Conclusions

This work presents a proof of concept for the high-speed readout electronics that will allow event-based SPAD arrays to be used in intensity-interferometry applications. Intensity interferometry is expected to be a major breakthrough for astronomy since it will significantly increase the spatial resolution of current telescopes. A two-stage design methodology was followed: prototyping of a software-based solution on a Raspberry Pi 5 SBC, and implementing a high-performance hardware-based acquisition system on an AMD Kria KR260 MPSoC platform. This methodology allowed for rapid development since both software and hardware platforms shared the same conditioning electronics. The result was a completely functional system operating at 100 MHz with asynchronous acquisition through the AER protocol used by the SPAD array. Experimental validation on an optical bench confirmed the system’s ability to detect and localize photonic events under different illumination conditions, with a median dark-count rate of 1.68 Hz at 27.8 °C and the majority of pixels being below 10 Hz. The integrity of communications via the AER protocol was verified using AMD’s ILA core, which was also used to evaluate the correctness of the transfer of events to the MPSoC’s PS memory via DMA. From that memory, events were sent via Ethernet to the processing and visualization computer. The FPGA design presents a WNS of 2.219 ns on the 100 MHz clock, equivalent to a maximum achievable frequency of about 128 MHz, ensuring sufficient margin for future extensions.

The prototype described in this paper demonstrates the technical feasibility of adapting event-based SPAD arrays, initially developed for LiDAR and 3D imaging, to photon-counting acquisition for astronomical intensity interferometry, laying the acquisition-electronics groundwork on which the timestamped, multi-telescope photon-correlation measurements planned as future work will be obtained. In future work, migration to a bare-metal implementation is planned to reduce the latency introduced by the PYNQ environment, along with the integration of the White Rabbit protocol for sub-nanosecond synchronization between telescopes, distributing a common disciplined clock and pulse-per-second (PPS) signal to each node and combining it with a per-event time-to-digital converter (TDC) for local sub-period interpolation, targeting resolutions on the order of 500 ps (Section 4.4). We also plan to incorporate the FPGA’s multigigabit transceivers to allow for high-speed communication (>10 Gbps) between the readout electronics and the data-processing computer via the Aurora 64B/66B protocol. This will also require the addition of an FPGA board to the computer via a PCIe slot. The proposed architecture lays the groundwork for the final deployment on five telescopes of the Roque de los Muchachos Observatory within the LPQI project. In parallel, the limitations characterized in this work, namely, the fill factor, the AER bus throughput ceiling, and the inter-pixel coupling observed in the dark-count map, are already informing the design of a next-generation SPAD sensor and its associated control electronics tailored to the LPQI project, so the results reported here feed directly into this ongoing effort. Beyond this specific deployment, the presented architecture, characterization results, and identified bottlenecks provide a reusable reference for adapting commercially or industrially developed SPAD arrays to other photon-starved scientific applications that demand asynchronous, high-throughput acquisition.

## Figures and Tables

**Table 1:** Main specifications of the 64 × 64 SPAD array used in this work.

Specification	Value
Technology	LFoundry 110 nm
Pixels	64 × 64
Readout	Asynchronous (event-driven)
Pixel pitch	24.5 μm
Fill factor	3.5%
Spectral range	400–800 nm
PDP	75% @ 425 nm
Power consumption	20.5 mW @ 10 Meps
Max. output event rate	40 Meps ^1^
Photon-accumulation latency (DVS mode, 128 photons)	1 μs ^2^

^1^ The sensor’s on-chip AER arbitration ceiling. The readout chain reported here has been measured at up to ≈124 keps under laboratory illumination (Section 4.3), a value well below this ceiling. ^2^ Minimum time to accumulate 128 photons in DVS (event-count) mode, limited by the ∼1 μs SPAD dead time [2]. This photon-collection latency is unrelated to the sub-nanosecond, per-event timestamp resolution targeted as future work via White Rabbit and an on-chip TDC (Section 4.4).

**Table 2:** Comparison with dedicated readout electronics reported for photon-counting stellar intensity interferometry. The systems target different design points—a small number of individually wired, timestamped channels versus a spatially multiplexed pixel array sharing one acquisition bus—so the rate figures are not directly comparable across that axis; see the discussion above.

	This Work	ASTRI Mini-Array [37]	On-Sky HPD Interferometer [38]
Detector	64 × 64 SPAD array	4-pixel SiPM	2-channel HPD
Readout topology	Spatially multiplexed, single shared AER bus	Individually wired channels	Individually wired channels
Highest rate tested	≈124 keps aggregate ^1^	up to 80 Mcps aggregate (4 ch.)	≈3.1 Meps (Vega, single detector) ^2^
Dark-count rate tested	≈1.68 Hz per pixel (median) ^3^	Not reported	≈50 Hz ^2^
Per-channel/pixel budget	≈1.63 keps per pixel (handshake-implementation ceiling; Section 4.4)	Not bus-shared	Not bus-shared
Per-event timestamping	Not yet implemented (future work)	Yes, ≈10 ps channel-to-channel skew	Yes, 42.4 ps FWHM (at ≈2 Meps)

^1^ Under the highest-illumination laboratory condition tested (Section 4.3), not a systematic saturation sweep (Section 4.4). ^2^ For [38], the highest-rate figure is the median single-detector photon count rate recorded for the brightest star observed (Vega), not a systematic saturation sweep. The dark-count figure corresponds to the detector bias and discrimination-threshold settings used in that work, not a swept characterization. ^3^ Median per-pixel dark-count rate at 27.8 °C (Section 4.3). This is not directly comparable in absolute terms to a per-channel SiPM dark-count rate, since one SiPM channel typically aggregates the dark-count contribution of many microcells over a photosensitive area much larger than one 24.5 μm SPAD pixel, and no per-channel or per-microcell figures for ASTRI’s SiPM array were reported in [37].

**Table 3:** FPGA resource utilization on the AMD Kria KR260.

Resource	Used	Available	Utilization (%)
LUT	11,579	117,120	9.89
FF	16,198	234,240	6.92
BRAM	114.50	144	79.51
IO	32	189	16.93

**Table 4:** Optical bench components used for experimental validation (Figure 6), all supplied by Thorlabs, Inc. (Newton, NJ, USA).

Component	Part Number
Fiber collimator	SM1SMA
Multimode fiber patch cable	M14L01
Achromatic doublet lens, f=200 mm	AC254-200-AB-ML
Achromatic doublet lens, f=150 mm	AC254-150-AB-ML
Adjustable iris diaphragm	SM1D12
30 mm cage plate	CP33/M
Cage assembly rod, 24″ long	ER24
Cage plate mounting base	CPB1/M
SM1 coupler	SM1T3

**Figure A1:**
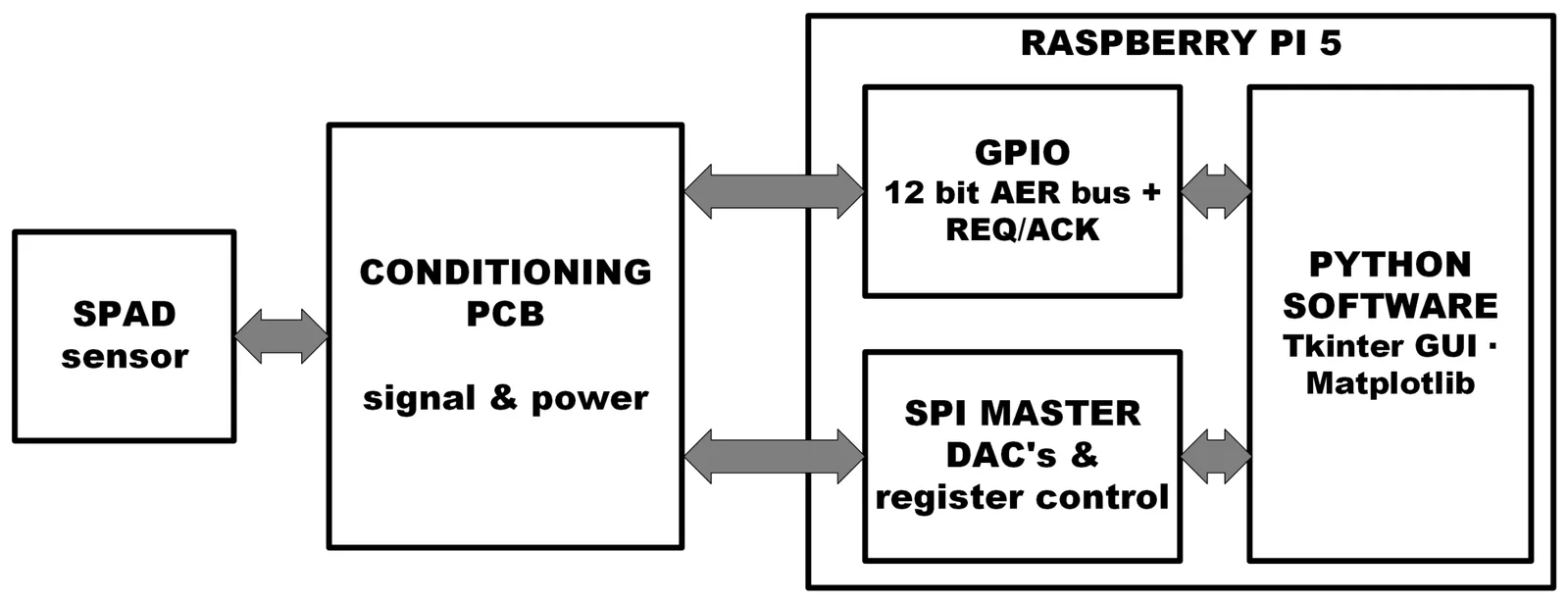
Block diagram of the Raspberry Pi prototyping stage. The 12-bit AER bus and the REQ/ACK handshake are handled through GPIO, while sensor configuration is performed over SPI; both feed the Python control and visualization software.

## Data Availability

The data presented in this study are available from the corresponding author on request.

## Abbreviations

The following abbreviations are used in this manuscript:

AER	Address-Event Representation
AXI	Advanced Extensible Interface
BRAM	Block RAM
COTS	Commercial, off-the-Shelf
DAC	Digital-to-Analog Converter
DCR	Dark-Count Rate
DMA	Direct Memory Access
DNW	Deep N-Well
DVS	Dynamic Vision Sensor
FF	Flip-Flop
FIFO	First-in First-out
FPGA	Field-Programmable Gate Array
FWHM	Full Width at Half Maximum
GPIO	General-Purpose Input/Output
GUI	Graphical User Interface
HPD	Hybrid Photon Detector
ILA	Integrated Logic Analyzer
IO	Input/Output
LDO	Low-Dropout (regulator)
LPQI	La Palma Quantum Interferometer
LUT	Look-Up Table
MPSoC	Multiprocessor System-on-Chip
PCB	Printed Circuit Board
PDP	Photon Detection Probability
PL	Programmable Logic
PPS	Pulse-per-Second
PS	Processing System
RMS	Root Mean Square
S2MM	Stream-to-Memory-Mapped
SBC	Single-Board Computer
SNR	Signal-to-Noise Ratio
SPAD	Single-Photon Avalanche Diode
SPI	Serial Peripheral Interface
TDC	Time-to-Digital Converter
TDL	Tapped Delay Line
ToF	Time-of-Flight
WNS	Worst Negative Slack
WR	White Rabbit

## Appendix A. Raspberry Pi Prototyping Stage

In the first stage, a Raspberry Pi 5 development platform is used as the main control and acquisition unit (Figure A1). The SPAD sensor operates in Free-Running mode, emitting events continuously. These events are transmitted to the Raspberry Pi through the conditioning PCB via the AER protocol, using a 12-bit bus (6 bits per X and Y coordinate) handled by GPIO, with REQ/ACK handshake signals driven by software. Sensor configurations such as bias, gain levels, and DAC values are set over the SPI protocol. The control software is written in Python and includes a Tkinter-based graphical user interface (GUI) that allows commencement and cessation of acquisition, selection of the operating mode, control of the sensor configuration registers, and visualization of pixel activity in real time through histograms and heat maps with matplotlib.

The main limitation of this stage is that the AER handshake is handled entirely in software, which restricts the maximum capture rate to a few kHz, well below the sensor’s capability. This stage is therefore exploratory and intended for functional validation rather than maximum-throughput acquisition. Importantly, the AMD Kria KR260 platform selected for the second stage features a 40-pin connector with the same physical layout as the Raspberry Pi 5, which allowed the conditioning PCB designed in this first stage to be reused directly, greatly simplifying the transition between platforms.
